# Guillain-Barré syndrome following the 2009 pandemic monovalent and seasonal trivalent influenza vaccination campaigns in Spain from 2009 to 2011: outcomes from active surveillance by a neurologist network, and records from a country-wide hospital discharge database

**DOI:** 10.1186/s12883-016-0598-z

**Published:** 2016-05-21

**Authors:** Enrique Alcalde-Cabero, Javier Almazán-Isla, Fernando J. García López, José Ramón Ara-Callizo, Fuencisla Avellanal, Carlos Casasnovas, Carlos Cemillán, José Ignacio Cuadrado, Jacinto Duarte, María Dolores Fernández-Pérez, Óscar Fernández, Juan Antonio García Merino, Rosa García Montero, Dolores Montero, Julio Pardo, Francisco Javier Rodríguez-Rivera, María Ruiz-Tovar, Jesús de Pedro-Cuesta, Spanish GBS Epidemiology Study Group

**Affiliations:** National Centre for Epidemiology, CIBERNED, Carlos III Health Institute, Madrid, Spain; Neurology Department, Miguel Servet University Hospital, Zaragoza, Spain; Neuromuscular Unit, Neurology Department, Bellvitge University Hospital, Bellvitge, Biomedical Research Institute (Institut d’Investigació Biomèdica de Bellvitge/IDIBELL), L’Hospitalet de Llobregat, Spain; Neurology Department, Severo Ochoa University Hospital, Leganés, Madrid Spain; Epidemiology Department, Regional Ministry of Health, Madrid Autonomous Region, Spain; Neurology Department, General Hospital, Segovia, Spain; Neurology Department, Virgen de las Nieves University Hospital, Granada, Spain; Neurology Department, Carlos Haya University Hospital, Málaga, Spain; Neurology Department, Puerta de Hierro University Hospital, Majadahonda, Madrid Spain; Neurology Department, Virgen de la Salud Hospital, Toledo, Spain; Spanish Medicines & Medical Devices Agency (Agencia Española de Medicamentos y Productos Sanitarios), Madrid, Spain; Neurology Department, University Teaching Hospital Clínico, Santiago de Compostela (Corunna), Spain; Neurology Department, La Paz University Hospital, Madrid, Spain

**Keywords:** Guillain-Barré syndrome, Influenza A virus H1N1 subtype, Influenza vaccines, Public health surveillance, Safety, ICD-9-CM

## Abstract

**Background:**

Studies have shown a slight excess risk in Guillain-Barré syndrome (GBS) incidence associated with A(H1N1)pdm09 vaccination campaign and seasonal trivalent influenza vaccine immunisations in 2009–2010. We aimed to assess the incidence of GBS as a potential adverse effect of A(H1N1)pdm09 vaccination.

**Methods:**

A neurologist-led network, active at the neurology departments of ten general hospitals serving an adult population of 4.68 million, conducted GBS surveillance in Spain in 2009–2011. The network, established in 1996, carried out a retrospective and a prospective study to estimate monthly alarm thresholds in GBS incidence and tested them in 1998–1999 in a pilot study. Such incidence thresholds additionally to observation of GBS cases with immunisation antecedent in the 42 days prior to clinical onset were taken as alarm signals for 2009–2011, since November 2009 onwards. For purpose of surveillance, in 2009 we updated both the available centres and the populations served by the network. We also did a retrospective countrywide review of hospital-discharged patients having ICD-9-CM code 357.0 (acute infective polyneuritis) as their principal diagnosis from January 2009 to December 2011.

**Results:**

Among 141 confirmed of 148 notified cases of GBS or Miller-Fisher syndrome, Brighton 1–2 criteria in 96 %, not a single patient was identified with clinical onset during the 42-day time interval following A(H1N1)pdm09 vaccination. In contrast, seven cases were seen during a similar period after seasonal campaigns. Monthly incidence figures did not, however, exceed the upper 95 % CI limit of expected incidence. A retrospective countrywide review of the registry of hospital-discharged patients having ICD-9-CM code 357.0 (acute infective polyneuritis) as their principal diagnosis did not suggest higher admission rates in critical months across the period December 2009-February 2010.

**Conclusions:**

Despite limited power and underlying reporting bias in 2010–2011, an increase in GBS incidence over background GBS, associated with A(H1N1)pdm09 monovalent or trivalent influenza immunisations, appears unlikely.

## Background

Guillain-Barré syndrome (GBS) is an acute, acquired immune-mediated polyradiculoneuropathy with mainly motor symptoms, preceded in two thirds of cases by an influenza-like or respiratory tract infection (ILI-RTI) or a gastrointestinal tract infection (GTI). GBS presents with an annual incidence of 1-2/100,000 in Western countries. Although immunotherapy with intravenous gammaglobulin (IVGG) considerably reduces mortality, GBS may cause severe disability [1, 2].

An association between influenza vaccines and GBS was previously described in 1976 and again in 1992–1994 [3–5]. Worldwide research, mainly using self-controlled case series design [6] and self-controlled risk-interval design [7, 8], has addressed potential increases in GBS incidence after vaccination campaigns against the 2009 pandemic influenza A(H1N1). Three meta-analyses, one including US data obtained from six adverse event monitoring systems [9], a pooled analysis across databases from 15 countries all over the world [10], and a meta-analysis of 16 published reports [11] showed a 2–3 fold excess risk in GBS incidence associated with A(H1N1)pdm09 vaccines, both adjuvanted and non-adjuvanted, when compared to no vaccination. However, other cohort studies failed to support this association [12–14] or yielded undetermined findings [15]. Although only a US study assessed the potential increase over background GBS incidence following seasonal or A(H1N1)pdm09 influenza vaccines, with negative results[16], it remains to be seen if occurrence of GBS attributed to A(H1N1)pdm09 vaccination exceeded previous seasonal and monthly GBS estimates.

This study sought to assess occurrence of GBS as a potential adverse effect of A(H1N1)pdm09 or seasonal vaccinations by means of a special surveillance system established to monitor GBS incidence during the vaccination programmes.

## Methods

This study consisted of two parts, namely: a prospective GBS surveillance system related to influenza vaccination, and a retrospective assessment of GBS incidence in Spain. In both parts, we attempted to detect incidence rates that were higher than previously reported.

### A(H1N1)pdm09 vaccine campaigns

In Spain, influenza vaccination is offered free of charge each year to people in high-risk groups, those over 6 months old with chronic conditions, elderly people over the age of 60 or 65 years depending on the region, healthcare workers, workers in essential public services, and caregivers. The A(H1N1)pdm09 vaccine was not recommended for elderly people without chronic disease.

#### 2009 seasonal trivalent influenza vaccine

The vaccine included A/Brisbane/59/2007(H1N1), A/Brisbane/10/2007(H3N2) and B/Brisbane/60/2008 strains. In the 2009 campaign, 10.2 million doses were administered country-wide between weeks 40 to 48.

#### 2009–2010 pandemic vaccine

The vaccine included the A/California/07/2009 (H1N1) virus strain. The vaccine brands were Focetria® (Novartis), adjuvanted with MF59, recommended to children and the elderly, Pandemrix® (Glaxo SmithKline), adjuvanted with ASO3, recommended for adults, and Panenza® (Sanofi Pasteur), unadjuvanted, recommended for pregnancy. Actual exposure involved administration of 970,468 doses in the second half of November 2009, 641,829 in December 2009, 114,220 in January 2010, coming to an end in late February 2010, making a total of 1.7 million doses, less than 5 % of which were administered to persons under 18 years of age. In all, 12 % of health care workers, 9 % of workers in essential public services, 15 % of people from 18 to 60 years old with chronic conditions, and 28 % of people 60 years old and over received the vaccine (Division of Vaccination Programmes, Spain’s Ministry of Health, Social Services, and Equality, unpublished report).

#### 2010 and 2011 campaigns

The vaccines included the A/California/07/2009 (H1N1), A/Perth/16/2009 (H3N2) and B/Brisbane/60/2008 strains [17, 18], a total of 8.1 million doses in each campaign.

In terms of vaccine effectiveness, estimates in Spain suggest that the 2009-TIV had no protective effect, A(H1N1)pdm09 vaccine had a good effect (66–78 %) and 2010-2011TIVs had a lower effect (50–55 %) [17–19].

### Neurologist network

An 11-hospital neurologist network, conceived as a special surveillance system [20], was established in 1996 to set alarm thresholds in GBS monthly incidence, so that incidence monitoring could be undertaken in situations where risk was perceived. The National Centre for Epidemiology at the Carlos III Institute of Health in Madrid co-ordinated the network, which then covered an adult population of 3.9 million. People under 20 years were not covered. The network incorporated epidemiological features of GBS in adults from 1985 to 1997 to estimate upper limits in monthly incidences, and then conducted a 2-year pilot prospective study to update upper thresholds in 1998–1999. As a result, the network estimated curves with expected monthly incidences for all and certain age groups [21, 22].

#### Surveillance design

At the request of the Spanish Agency of Medicines and Medical Devices (*Agencia Española de Medicamentos y Productos Sanitarios/AEMPS*), the National Centre for Epidemiology, acting as a Central Unit, contacted network members in August 2009 and re-established the network to detect a potential GBS outbreak related to A(H1N1)pdm09 vaccination. The eleven hospitals constituting the original network remained functionally appropriate for surveillance with minor changes in referral, staff and catchment population but one hospital which had difficulties in notifying cases was excluded. In Spain, a national health service covers the majority of population. To estimate incidence rates, each participant hospital updated the number of people it covered. Of a total of 36.9 million Spanish residents aged ≥20 years, the population under surveillance numbered 4.68 million, approximately 1/8 of the country population of that age. Prospective notification to the Central Unit of all patients suspected of presenting with GBS started in September 2009, approximately one month before onset of the A(H1N1) monovalent vaccine campaign, and ended on 31 December 2011. Retrospective reporting dated back to 1 January 2009. The Central Unit was tasked with identifying potential outbreaks on the basis of the information supplied by local neurologists, and, when applicable, notifying these to the *AEMPS*.

#### Study subjects

A GBS case was defined by reference to the National Institutes of Neurological Disorders and Stroke (NINDS) criteria [23]. Two months after notification of a suspected case of GBS, local neurologists confirmed or excluded the diagnosis on the basis of clinical, neurophysiological or cerebrospinal fluid parameters. Miller-Fisher syndrome (MFS) was not compulsorily reported. Each GBS or MFS case was re-classified at the end of the 36-month study by one local neurologist and JdP-C, at the Central Unit, using the recent and prevailing Brighton Collaboration case-definition categories [23, 24].

The *AEMPS* checked the presence of other possible GBS cases reported as suspected adverse drug reactions in the population covered by network hospitals. For data-completion purposes, in 2012 we requested all hospital pharmacies to provide information on registered IVGG deliveries to wards for treatment of GBS patients admitted during the period 2009–2012. These requests did not add any new patient.

#### Clinical antecedents

We collected information on immunisations, including influenza vaccines, administered during the 42-day period preceding clinical onset of suspected GBS. For other clinical antecedents, we only investigated those occurring during the 30-day period prior to clinical onset of suspected GBS, namely, respiratory infections (fever with cough or expectoration and other respiratory symptoms), gastrointestinal infections (nausea, vomiting, diarrhoea), other infections, and other antecedents. We collected antecedents through interviews with patients. The *AEMPS*, working in collaboration with the public health authorities, would certify the vaccine received (brand, health centre where the prescription was issued, and administration date) in A(H1N1)pdm09 post-vaccination cases.

### Database of hospital discharges

At the end of 2013, we requested the National Hospital In-patient Registry to supply us with data on all patients aged ≥20 years admitted in the period 2009–2011 and having International Classification of Diseases, Ninth Revision, Clinical Modification (ICD-9-CM) code 357.0 (acute infective polyneuritis) as their principal or other diagnosis at discharge. This registry constitutes a database on discharges generated at all hospitals serving the Spanish National Health Service, which provides services for the large majority of the resident population country-wide and encompasses all hospitals in the surveillance network [25].

### Ethical approval

This study was approved by the Research Ethics Committee at the Carlos III Institute of Health. All patients, with no exceptions, gave their informed consent.

### Statistical analysis

We computed exact Poisson confidence intervals (CI) for observed incidence rates for all, age groups, and genders [26]. We accepted as valid the background incidence and threshold values obtained from the previous study in 1998–1999, despite a possible change in hospital admissions due to the widespread use of IVGG as early treatment for GBS, including mild cases. We defined alarm signals when post A(H1N1)pdm09 immunisation cases were notified by the network or the total monthly incidence exceeded the estimated monthly upper 95 % CI limit for background incidence [22]. Monthly upper 95 % CI limit for background incidence had been calculated with ARIMA and Poisson models [21]. Crude monthly incidences were calculated from notifications and confirmed cases, and plotted on a graph showing predicted values.

We checked the positive predictive value of ICD-9-CM diagnostic code 357.0 in the Hospital In-patient Registry against the judgment of a neurologist (JAG-M) at one of the hospitals, the Puerta de Hierro University Hospital in Majadahonda. For each centre, we estimated a sensitivity value as the complementary of the false-negative rate, defined as the ratio between the number of notified and confirmed GBS cases not registered at the National Hospital In-patient Registry over the total number of notified and confirmed GBS cases. We calculated monthly incidences of hospital-admitted GBS country-wide and plotted these on the epidemic curve defined by the 95 % CI limits of expected incidences depicted in the above-mentioned graph.

## Results

### GBS cases ascertained by the neurologist network

**Fig. 1:**
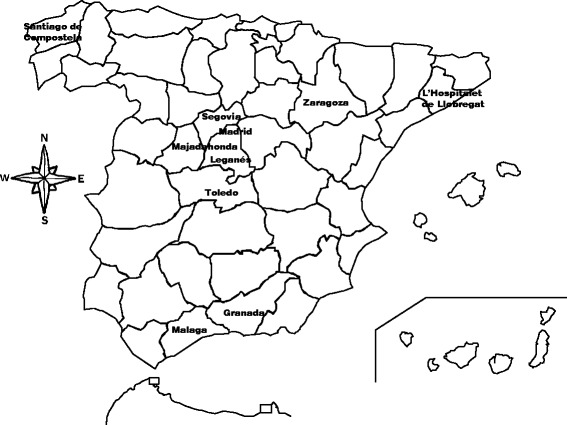
Geographical distribution of cities where study hospitals provided neurological care for Guillain-Barré syndrome. The inset shows Canary Islands. This figure is slightly modified from Cuadrado et al. [22]. Copyright 2004, with permission of Springer

Figure 1 shows the geographical distribution of the 10 hospitals in the network. During the surveillance period, January 2009-December 2011, local neurologists notified 148 patients with suspected GBS to the Central Unit. Of these, 138 were confirmed as GBS and three as MFS, making a total of 141 diagnoses confirmed by reference to the NINDS criteria after a minimum follow-up of one month. The positive predictive value of a suspected or notified case was 141/148, 95 %, 95 % CI 90 % to 98 %. When patients were re-classified according to Brighton criteria, 81 (57 %), 54 (38 %), and 5 (4 %) met criteria for levels 1, 2 and 3, respectively; one case (0.7 %) remained unclassified due to lack of access to hospital records. Of the seven patients excluded from the initially reported total of 148, three had alternative neurological diagnoses and four had a doubtful GBS diagnosis.

Of the 141 cases, 82 (58 %) were men; mean age at onset was 55.9 years (standard deviation 17.28 years); and 48 patients (34 %) were bed-bound one week after onset (Table 1). Two patients with serious comorbidity died within the first week of onset and a further four at a later date.

**Table 1:** Characteristics of confirmed GBS cases notified in 2009–2011 and identified by the neurologist network

		Motor status at one week after clinical onset or hospital admission				
Variables	No. of patients (%)	Independent gait	Gait: able with support	Able to stand up	Bed-bound	Unknown
Sex						
Male	82 (58.2)	21	21	3	31	6
Female	59 (41.8)	18	22	0	17	2
Age group (years)						
20–29	11 (7.8)	5	4	0	1	1
30–39	23 (16.3)	14	6	0	2	1
40–49	21 (14.9)	5	9	1	5	1
50–59	25 (17.7)	4	13	1	6	1
60–69	24 (17.0)	6	5	0	12	1
70–79	26 (18.4)	2	4	0	17	3
80+	11 (7.8)	3	2	1	5	0
Clinical antecedent						
Not identified	41 (29.1)	14	13	1	9	4
Recorded	100 (70.9)	25	30	2	39	4
Infection	85 (60.3)	21	27	2	32	3
—GTI	40 (28.4)	9	13	1	15	2
—ILI-RTI	34 (24.1)	11	9	1	12	1
—GI and ILI-RTI	3 (2.1)	0	1	0	2	0
—Urinary tract	3 (2.1)	0	2	0	1	0
—Other	4 (2.8)	1	2	0	1	0
—Unknown	1 (0.7)	0	0	0	1	0
Influenza vaccination	7 (5.0)	2	1	0	4	0
Other^a^	13 (9.2)	2	4	0	6	1
All patients	141^a,b^	39 (27.7)	43^b^ (30.5)	3 (2.1)	48^b^ (34.0)	8 (5.7)

*GBS* Guillain-Barré syndrome, *GTI* gastrointestinal tract infection, *ILI-RTI* influenza-like infection or respiratory tract infection

^a^“Other” encompasses surgery, medication, trauma, gastric carcinoma, delivery and pregnancy

^b^The sum does not add up the total amount of patients because some patients had more than one antecedent

### GBS incidence according to the neurologist network

Table 2 shows the age- and sex-specific distribution of cases and incidences. The annual incidence per 100,000 adult population was 1.20 for men and 0.81 for women. Incidence increased with age, particularly among men, up to the 70–79 age group. Table 3 shows the distribution of cases and incidence by hospital. Average annual incidence per 100,000 ranged from 0.35 in Malaga to 2.51 in Santiago de Compostela.

**Table 2:** Observed incidence of GBS per 100,000 person-years for 2009–2011 in the population of 4.68 million under surveillance by the neurologist network

	Male				Female				Total			
Age group in years	Cases	Person-years	Incidence	95 % CI	Cases	Person-years	Incidence	95 % CI	Cases	Person-years	Incidence	95 % CI
20–29	6	1,232,652	0.49	(0.18–1.06)	5	1,224,264	0.41	(0.13–0.95)	11	2,456,916	0.45	(0.22–0.80)
30–39	12	1,607,871	0.75	(0.39–1.30)	11	1,560,600	0.70	(0.35–1.26)	23	3,168,471	0.73	(0.46–1.09)
40–49	14	1,350,147	1.04	(0.57–1.74)	7	1,354,077	0.52	(0.21–1.07)	21	2,704,224	0.78	(0.48–1.19)
50–59	10	991,665	1.01	(0.48–1.85)	15	1,045,449	1.43	(0.80–2.37)	25	2,037,114	1.23	(0.79–1.81)
60–69	17	783,156	2.17	(1.26–3.48)	7	845,904	0.83	(0.33–1.71)	24	1,629,060	1.47	(0.94–2.19)
70–79	14	569,067	2.46	(1.34–4.13)	12	712,188	1.68	(0.87–2.94)	26	1,281,255	2.03	(1.33–2.97)
80+	9	272,355	3.30	(1.51–6.27)	2	503,019	0.40	(0.05–1.44)	11	775,374	1.42	(0.71–2.54)
Total ≥20 years	82	6,806,913	1.20	(0.96–1.50)	59	7,245,501	0.81	(0.62–1.05)	141	14,052,414	1.00	(0.84–1.18)

*GBS* indicates Guillain-Barré syndrome, *CI* confidence interval

**Table 3:** Hospitals and population coverage in numbers, GBS patients notified to the neurologist network, estimated sensitivity compared to patients coded as GBS in the National Hospital In-patient Registry and incidence × 100,000

Hospital	Person-years ≥20 years	Number of patients						Proportions		Incidence × 100,000	
		Number of hospital-registered patients^a^	Notified patients	Notified and confirmed GBS (a)	Confirmed not hospital registered (b)	Infection in preceding 30 days	Influenza vaccine in preceding 42 days	Confirmed not registered over notified and confirmed (b/a)	Estimated sensitivity 1-(b/a)	in registered patients^a^	in notified patients
Carlos Haya Hospital (Malaga)	3,676,698	19	13	13	4	8	0	0.31	0.69	0.52	0.35
General Hospital (Segovia)	364,983	6	4	4	0	3	0	0.00	1.00	1.64	1.10
La Paz University Hospital (Madrid)	1,239,486	27	19	18	5	10	2	0.28	0.72	2.18	1.45
Miguel Servet University Hospital (Zaragoza)	1,023,261	13	11	9	1	3	0	0.11	0.89	1.27	0.88
Bellvitge University Hospital (Hospitalet de Llobregat)	3,034,845	31	20	20	7	11	2	0.35	0.65	1.02	0.66
Puerta de Hierro University Hospital (Majadahonda)	967,029	17	15	14	0	8	0	0.00	1.00	1.76	1.45
Severo Ochoa University Hospital (Leganés)	472,563	8	5	5	0	3	0	0.00	1.00	1.69	1.06
Virgen de la Salud Hospital (Toledo)	1,003,170	16	17	16	6	11	0	0.38	0.63	1.59	1.59
Virgen de las Nieves University Hospital (Granada)	1,115,721	18	15	13	4	8	0	0.31	0.69	1.61	1.17
University Hospital Clínico (Santiago de Compostela)	1,154,658	38	29	29	8	20	3	0.28	0.72	3.29	2.51
Total	14,052,414	193	148	141	35	85	7	0.25	0.75	1.37	1.00

*GBS* Guillain-Barré syndrome

^a^Patients admitted to the respective hospitals during the period 2009–2011 and discharged with ICD-9-CM code 357.0 as their principal diagnosis

### GBS cases registered at the National Hospital In-patient Registry

A total of 2383 patients were hospital-admitted country-wide from 2009 to 2011 with ICD-9-CM code 357.0 as their principal diagnosis. The number of registered patients with GBS admitted to the networked hospitals across the period 2009–2011 was 193, 37 % higher than the 141 notified and confirmed GBS and MFS cases, with a several-fold variation in incidence among hospitals. Thirty-five out of the 141 patients with confirmed GBS reported by surveillance from 2009 to 2011 (25 %) were not identified among those with GBS code 357.0 as their principal diagnosis (sensitivity 75 %, 95 % CI 67 % to 82 %). Likewise, the neurologist network reported 106 out of the 193 patients who were registered as admitted from 2009 to 2011 to networked hospitals and discharged with code 357.0.

The validation of ICD-9-CM code 357.0 for GBS diagnosis, recorded for 17 patients at the Puerta de Hierro Hospital in Majadahonda, showed it to be wrong in three cases, 18 %, which had in fact been diagnosed with axonal polyneuropathy, chronic transformation of a previous GBS and non-inflammatory polyradiculoneuropathy attributable to causes other than GBS. Hence, the positive predictive value of such 357.0 coded diagnosis was 82 %, 95 % CI 57 % to 96 %.

### Vaccination and other antecedents

One hundred cases (71 %) had a record of potential exposures (Table 1), with 85 (60 %) patients having had infections, 7 (5 %) having received immunisations with influenza vaccine within the 42 days prior to clinical onset and 13 (9 %) having other antecedents.

Table 4 shows demographic and clinical data for the seven cases with vaccination, who were admitted to three different hospitals. All cases fulfilled Brighton level-1 criteria. Four had received a 2009-TIV prior to A(H1N1)pdm09 campaign, and one of these was simultaneously affected by a bronchopulmonary infection as a clinical antecedent. None had received A(H1N1)pdm09 vaccine. Another three cases had received a 2010–2011-TIV immunisation containing the A(H1N1)pdm09 strain, one in the 2010 campaign and two in 2011. Four out of seven GBS cases preceded by influenza vaccination were bed-bound compared with 43 of 134 other GBS cases (Tables 1 and 4).

**Table 4:** Neurologist network: demographic and clinical data pertaining to seven GBS patients with influenza immunisations during the 42-day period preceding clinical onset

Patient ref. no., sex, age	Vaccination date and type (a)	Co-morbidity or clinical antecedent in 30 days prior to onset	Symptom onset (b)	(a-b) Interval in weeks	Functional level at one week	Treatment	Clinical confirmation on the basis of
1 Female 53	2 Oct 2009	Motor neurone disease	13 Oct 2009	1–2	Walking	Not treated	Clinical symptoms/exam.
	2009-TIV						Cerebrospinal fluid (CSF) tests
							Electrophysiology lab.
							Other causes excluded
2 Male 68	27 Oct 2009	Heart disease.	22 Nov 2009	3–4	Bed-bound	IVGG	Electrophysiology lab.
	2009-TIV	Sleep apnea. High blood pressure. Bronchopulmonary infection					Other causes excluded
3 Male 34	20 Oct 2009	Syphilis seropositive	30 Nov 2009	5–6	Walking	IVGG	Clinical symptoms/exam.
	2009-TIV						CSF tests
							Electrophysiology lab.
							Other causes excluded
4 Male 70	28 Sept 2009	Motor neurone disease	10 Oct 2009	1–2	Bed-bound	IVGG	Clinical symptoms/exam
	2009-TIV						CSF tests
							Electrophysiology lab.
							Other causes excluded
5 Male 77	18 Oct 2010	-	1 Nov 2010	2	Bed-bound	IVGG	Clinical symptoms/exam.
	2010–2011-TIV^a^						Electrophysiology lab. Other causes excluded
6 Female 78	5 Jan 2011	-	15 Jan 2011	1–2	Able to walk with support	IVGG	Clinical symptoms/exam.
	2010–2011-TIV						CSF tests
							Electrophysiology lab. Other causes excluded
7 Male 82	27 Oct 2011	High blood pressure	23 Nov 2011	4	Bed-bound	IVGG Mechanical Ventilation	Clinical symptoms/exam.
	2010–2011-TIV^a^	DM					CSF tests
		Atrial fibrillation					Electrophysiology lab. Other causes excluded

*GBS* Guillain-Barré syndrome, *DM* diabetes mellitus type 2, *TIV* seasonal trivalent influenza vaccine, *IVGG* intravenous gammaglobulin

^a^Received the vaccine Chiromas® (Novartis), adjuvanted with MF59C.1

As no GBS case related to A(H1N1)pdm09 vaccination was found we did not send any alarm signal to health authority, the *AEMPS*.

**Fig. 2:**
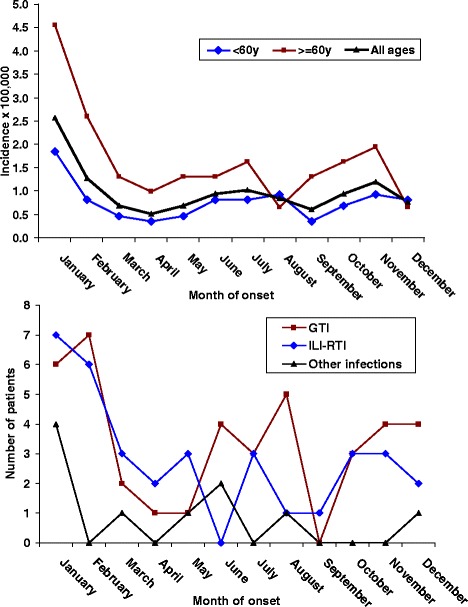
Seasonal patterns of Guillain-Barré syndrome (GBS) according to the neurologist network. Top: monthly incidence of GBS in two age groups. Bottom: case distribution by type of preceding infection and month of clinical onset

Figure 2 depicts the monthly/seasonal incidence of GBS in patients grouped by age and type of most frequent antecedent. The seasonal distribution suggested the highest incidence in January and February, particularly for patients aged ≥60 years and for those with previous ILI-RTI or GTI.

### 2009-2011 GBS incidence compared with background incidence

**Fig. 3:**
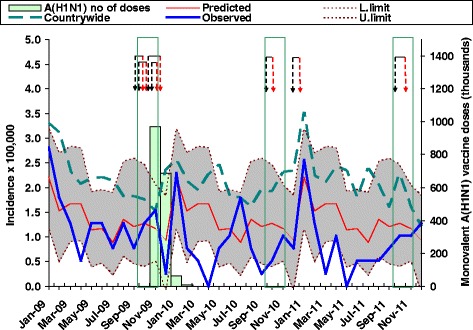
Predicted Guillain-Barré syndrome (GBS) background incidence with its 95 % confidence limits; monthly incidence of GBS among the population under surveillance as observed by the neurologist network during the period 2009–2010, seasonal and A(H1N1)pdm09 influenza vaccination campaigns (in the latter case with monthly number of doses for the whole population from 16 November 2009 to 1 February 2010, and routine campaign intervals —marked between vertical green lines—for seasonal immunisations), clinical antecedents (black arrows) and clinical onset of GBS patients (red arrows) immunised during the 42-day prior to clinical onset; and monthly incidence of GBS during the surveillance period, as seen from country-wide diagnostic data on 2383 hospital-admitted patients over 20 years and having GBS coded as ICD-9-CM 357.0 as their principal diagnosis at discharge (Discharge Certificates, National In-Patient Hospital Registry)

Figure 3 gives an overview of the incidence and antecedents during the surveillance period compared to the predicted background incidence. Monthly incidences of notified and confirmed GBS did not exceed the 95 % upper confidence limit but did exceed the lower limit several times in 2010 and 2011. GBS rates from National Hospital In-patient Registry were generally higher than those calculated from notified patients, particularly after the 2010-2011-TIV campaign, and occasionally exceeded the upper limit of the epidemic prediction, though not for the period October-November 2009 when the four post-2009-TIV cases were observed or the period following the A(H1N1)pdm09 vaccination campaign.

## Discussion

The performance of our surveillance of GBS as a potential adverse reaction of influenza immunisations rests on the coincidence of time-related clustering of GBS cases occurring within the 42-day, high-risk post-immunisation period with a greater-than-expected increase in GBS incidence. Overall, monthly incidence of GBS, whether notified or registered country-wide, does not appear to reflect any impact of A(H1N1)pdm09 or seasonal vaccine campaigns. Our neurologist network failed to find a single GBS case following A(H1N1)pdm09 vaccination. The Hospital In-patient Registry suggested no increase in GBS monthly incidence during or post influenza immunisation campaigns.

Our study might have some limitations. The power of this study to detect an increase in incidence in vaccinated persons depends on the method of determination of the threshold, on the relative risk of GBS in vaccinated persons, and on the proportion of the population that received the vaccine. Since only a small part of the population was vaccinated with A(H1N1)pdm09, the relative risk associated with vaccination should have been very high to induce an overall incidence of GBS sufficiently high to exceed the threshold. However, we did not find any GBS case following A(H1N1)pdm09 vaccination in the 165,000 population vaccinated, despite an expected background incidence around 2.62 cases from December to March, which makes increased incidence associated with vaccination highly unlikely. Another limitation is the difficulty in adjusting for confounding from causes such as ILI-RTI or GTI, both of which are unlikely in our series in the light of the lack of such antecedents in six of our seven cases. Furthermore, potential underreporting might have happened, as has been suggested in connection with paediatric and medical departments in Sweden, where a similar GBS surveillance system was in place [27]. In our study, only one reported GBS case was not hospital-admitted but similar cases may have been overlooked. Underreporting in autumn 2009, linked to considerable media attention paid to influenza-related events during the influenza monovalent vaccine campaign, was probably lower than during other surveillance periods. Additionally, National Hospital In-patient Register has a proportion of false positive and false negative cases whose impact on this registry’s ultimate validity for estimating all GBS cases is uncertain [28]. Likewise, we assumed that background incidence estimated in 1999 would remain the same 10 years later, despite the potential change in infection transmissibility caused by the increase in population density in Spain over this period. Neither can exposure misclassification be ruled out since recall bias might have affected vaccination ascertainment. Our study also lacks power to study the individual effects of every vaccine adjuvant. Lastly, one of the post-2009-TIV Spanish cases had preceding ILI-RTI episodes, and two others had motor function potentially compromised by motor neurone disease. One could thus speculate that co-morbidity may have contributed to vaccination being indicated. High immunisation frequencies and improved information might have mimicked excess post-2009-TIV GBS among the surveillance population in both Spain and the United Kingdom [29].

Our results are consistent with a study by Burwen et al. [16] on GBS incidence after influenza vaccination among the Medicare population in 2009–2010, which failed to find an increase in GBS incidence above a critical limit established by GBS incidence in the previous five years. In contrast, our results differ from the modest risk excess reported in three pooled analyses [9–11]. However, the main design of these reviews, namely, self-controlled case series analysis and self-controlled interval-risk design, might imply a high risk of bias, i.e., recall bias for exposures predating GBS onset by more than 42 days and, in particular, incomplete adjustment for potential confounders like ILI-RTI or GTI and seasonal patterns of GBS onset [30, 31]. On the other hand, cohort studies either give lower relative risks [11, 15, 32] or fail to find any association [12–14].

Contrary to the findings of the ITANG study, which used a 176-patient case–control and case-series designs and reported modest but positive associations with 2010-TIV [33], we failed to observe any suspected impact of either the 2010 or the 2011 seasonal immunisation. Doubts nevertheless continue to surround ITANG’s capacity to remove residual confounding from the several-fold higher effect of the preceding infections, odds ratio (OR) = 23.8 and OR = 11.5 for ILI-RTI or GTI, respectively. Even so, the most important reason for suspecting a high risk of bias in the ITANG study is control selection towards low 2010-2011-TIV immunisation rates in hospital controls. ITANG controls were recruited from among patients with trauma, after eliminating those with chronic conditions, a selection strategy that would potentially exclude people in whom 2010-2011-TIV had been indicated.

One important aspect of our results regarding the four cases associated with the 2009-TIV and not with preceding infections is that they might match the intriguing association observed in the UK for the same exposure and time as reported by the VAESCO study. The VAESCO study reported an excess GBS risk for the unadjuvanted 2009-TIV in the UK [34], not seen in previous years using the same UK database [29]. The fact that such an effect was not observed in other seasonal influenza vaccines in 2010 or 2011 in Spain might point to a side-effect of the 2009-TIV due either to the A/Brisbane/59/2007(H1N1) or to the A/Brisbane/10/2007(H3N2) component of the 2009 TIV being removed from the 2010-2011-TIV.

Our study design, when compared to the other three procedures used to monitor or disclose potential causes of GBS, i.e., the spontaneous reporting of suspected adverse drug reactions, self-controlled case series and case–control analysis, raises public health service-related questions about preferential or alternative procedures when facing early detection of a potential excess risk of GBS. It would appear that methodological issues and logistic elements related to the question to be answered are paramount. Speed in activating surveillance resources may constitute a key issue. Self-controlled case-series analysis in New York based on a list of 150 active-reporting neurologists and 2495 passive-reporting neurologists based in neurology departments, was considered expensive [35]. Our system, relying on hospital neurology departments, took advantage of the National Health Care Services which, on a geographical residential basis, covered well-defined populations, was underpinned by a small number of neurologists and was conducted at no extra cost. Spain’s National Health Care system is operated by regional authorities and the *AEMPS* is a state-owned institution. The Spanish neurologist network was intensively active during the A(H1N1)pdm09 campaign, i.e., though the low monthly rates in 2010 and 2011 might have been related to underreporting. Reduction of surveillance to the November 2009-March 2010 period or express official support from the regional authorities for the purpose of incorporating public health missions in neurology departments might have improved GBS surveillance.

It would appear that GBS case–control studies using population controls and embodied in a hospital- and population-based network may be the best alternative for populations covered by publicly operated medical systems. This option does not exclude the addition of other procedures, such as self-controlled GBS case series, whether or not nested within an immunised cohort. At all events, knowledge of the GBS epidemiology in the study population is advantageous, particularly because asymmetry of exposure and confounder measurement in non-high-risk periods or among controls appears to be inevitable. Although A(H1N1)pdm09 monovalent vaccine is no longer in use, its strain has been incorporated in the seasonal vaccines of the following years, so that our study results are still of practical significance. All things considered, an effective influenza vaccine might reduce GBS incidence through reductions in ILI-RTI infections, a far more frequent antecedent to GBS than is vaccination, as shown by our results.

## Conclusions

We conclude that increased GBS incidence due to A(H1N1)pdm09 or TIV immunisations during 2010–2011 over background GBS is unlikely. Efficient GBS surveillance requires the local support of public health authorities and methodological updates, in line with the designated purpose in each case.

### Ethics and consent to participate

This research was approved by the Comité de Ética en la Investigación y Bienestar Animal del Instituto de Salud Carlos III, with the reference number CEI PI 20_2009. All patients, with no exceptions, gave their informed consent.

### Consent to publish

Not applicable.

### Availability of data and materials

We will offer our surveillance database duly anonymised, upon request to the corresponding author. As for the cases of Guillain-Barré syndrome coded in the National Hospital In-patient Registry we do not have permission from the Spain’s Ministry of Health to share it with third parties.

## Abbreviations

A(H1N1)pdm09: pandemic A(H1N1)2009 virus
AEMPS: Spanish Agency of Medicines and Medical Devices (*Agencia Española de Medicamentos y Productos Sanitarios)*
CI: confidence interval
GBS: Guillain-Barré syndrome
GTI: gastrointestinal tract infection
ICD-9-CM: International Classification of Diseases, Ninth Revision, Clinical Modification
ILI-RTI: influenza-like or respiratory tract infection
IVGG: intravenous gammaglobulin
MFS: Miller-Fisher syndrome
NINDS: National Institutes of Neurological Disorders and Stroke
OR: odds ratio
TIV: seasonal trivalent influenza vaccine
